# Imperforate hymen and leaking hematosalpinx mimicking acute appendicitis: A report of a rare case and a review of literature

**DOI:** 10.1016/j.ijscr.2019.09.003

**Published:** 2019-09-18

**Authors:** Foster Amponsah-Manu, Paddy Ssentongo, Temitope Arkorful, Richard Ofosu-Akromah, Anna E. Ssentongo, Seth Hansen-Garshong, John S. Oh

**Affiliations:** a Department of Surgery, Eastern Regional Hospital, P.O. Box 201, Koforidua, Ghana; b Department of Surgery, Penn State Hershey College of Medicine and Milton S. Hershey Medical Center, Hershey, PA, USA; c Department of Public Health Sciences, Penn State Hershey College of Medicine and Milton S. Hershey Medical Center, Hershey, PA, USA; d Center for Neural Engineering, Department of Engineering, Science and Mechanics, Pennsylvania State University, University Park, PA, USA

**Keywords:** Imperforate hymen, Acute appendicitis, Hematocolpometra, Case report

## Abstract

**Introduction:**

Cases of imperforate hymen with leaking hematosalpinx into the peritoneum are rare. We present a case of imperforate hymen mimicking acute appendicitis creating diagnostic challenges with a near miss diagnosis.

**Case:**

A 12-year-old premenarchal female presented with severe pain that migrated from the epigastrium to the right lower quadrant. Physical examination revealed an ill-defined non-tender mass in the right lower quadrant on deep palpation, without signs of peritonitis. We made a diagnosis of an appendiceal mass and the patient underwent an appendectomy. We found approximately 200 mL of dark blood in the peritoneum and an inflamed appendix. After the appendectomy we noticed an engorged right fallopian tube leaking into the peritoneum and a bulky pelvic mass in the lower segment of the uterus. Perineal examination revealed a slightly bulging imperforate hymen. Hymenectomy was performed and 500 mL of viscous old blood was drained. On follow-up, the hymen was still open.

**Discussion:**

Imperforate hymen commonly presents with cyclic abdominal pain and enlarging mass in premenarchal females, it is rare to present with hemoperitoneum and mimicking acute appendicitis.

**Conclusion:**

Although rare, imperforate hymen with retrograde menstruation that causes dilatation of the vagina and uterus (i.e., hematocolpometra) is an important consideration in the differential diagnosis of abdominal pain in premenarchal females with abdominal pain. It is easily diagnosed by physical examination. However, if patient or parents refuse genital exam evaluation, imaging studies can greatly help with diagnosis. Ultrasound will show an echogenic fluid accumulation in the vagina that can extend to uterus.

## Highlights

• Cases of imperforate hymen (IH) that present with hemoperitoneum mimicking acute appendicitis are rare.
• Diagnosis and management can be a challenge especially in low-resource setting.
• Surgical management of both acute appendicitis and IH can be successful achieved during the same operative session.

## 1 Introduction

Imperforate hymen (IH), although rare, is the most common obstructive congenital anomaly of the female genital tract, with an incidence rate of approximately 1 in 2000 females [1]. Patients are usually asymptomatic until menarche when menstrual blood starts to collect behind the imperforate hymen, accumulating either in the vagina (hematocolpos) [2], uterus (hematometra) or/and fallopian tubes (hematosalpinx). Hematocolpos gets worse with each menstrual period. If IH is not surgically treated early, it may result in complications such as endometriosis, adhesions, and ultimately infertility.

The classical presentation of imperforate hymen is primary amenorrhea in adolescent girls with a bulging mass in the vagina, associated with cyclic abdominal pain [3]. Atypical presentation can include urinary retention with kidney failure [4], chronic constipation [5], and uterine infections due to static uterine blood. As a result, girls presenting with atypical IH often are initially misdiagnosed, which increases the likelihood of undergoing unnecessary and yet costly laboratory and radiographic studies. The aim of this case report is to present IH mimicking acute appendicitis. We further review the literature on the different unique presentations of imperforate hymen and discuss surgical management options. This work has been reported in line with the SCARE criteria [6].

## 2 Case

In March 2019, a 12-year-old premenarchal girl was referred to our hospital for the definitive management of acute appendicitis. She presented with a 5-day history of increasing abdominal pain. On admission to our hospital, the abdominal pain was located in the epigastrium and at the right lower quadrant (RLQ). The pain was gradual in onset and progressively worsened. The pain was severe, 8 on a scale of 1–10, sharp, constant but non-radiating. It was associated with constipation but not with anorexia, nausea or vomiting. The patient did not report any history of dysuria or any change in urinary frequency. She had unremarkable gynecological, birth and developmental history.

**Fig. 1:**
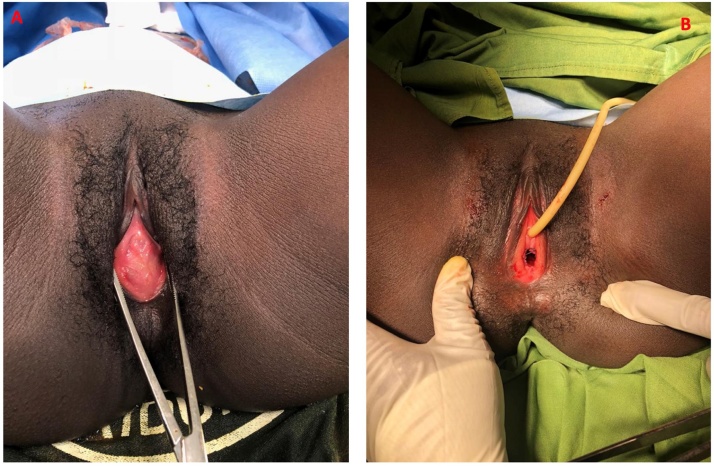
Imperfect hymen: A (before hymenectomy) B (After hymenectomy with a cruciate incision).

On examination, the only abnormal sign was an ill-defined non-tender mass in the RLQ on deep palpation. The abdomen was not distended and without signs of peritonitis. A perineal physical examination was missed to be carried out by the attending surgeon and the admitting surgical resident. (This was a failure on the surgical team and a very important lesson we learned to avoid in the future). Based on the clinical symptoms of RLQ pain, tender mass and the migratory nature of the abdominal pain, a differential diagnosis of an appendicitis was made. The patient was prepared for an appendectomy. Her lab work was within normal limits: Hb: 12.5 g/dL (normal range: 12.0–16.0 g/dL), WBC: 9400 cells/uL (normal range: 4000–10,000 cells/uL), Platelets: 319,0000 cells/uL (range: 100,000–300,000 cells/uL). Since the patient’s presentation was classic for appendicitis, no imaging studies were needed. The patient was hydrated with intravenous (IV) normal saline and was started on broad-spectrum IV antibiotics (metronidazole and ciprofloxacin). Under general anesthesia, a RLQ incision over the McBurney point (located two thirds of the distance between the umbilicus and the inferior superior iliac spine) was made. On entering the peritoneum, an inflamed appearing appendicitis was noted. There was approximately 200 mL of dark blood in the peritoneum. We performed an appendectomy. Blood in the peritoneum was drained and the abdomen was thoroughly irrigated with warm saline solution. During the inspection of the bowel, we noted a ruptured, hemorrhagic right ovarian follicle and a bulky pelvic mass in the lower segment of the uterus. Under general anesthesia, a vaginal examination was conducted, and a slightly bulging imperforate hymen was identified (Fig. 1A). A hymenectomy was performed through a cruciate incision followed up with re-approximation of hymen edges with Vicryl 2-0 sutures (Fig. 1B). More than 500 mL of viscous old blood was drained from the vagina, uterus and the right salpinx. The bleeding right ovarian follicle was sutured. Both ovaries were of normal size on palpation. She was then discharged on postoperative day 4 without pain or vaginal bleeding. A repeat genitourinary examination 3 weeks later still showed a patent hymen.

## 3 Discussion

Although imperforate hymen is an anomaly usually diagnosed by a thorough history taking and a physical examination, the presentation mimicking acute appendicitis and a tender mass in the RLQ was atypical. The appendix was secondarily inflamed by the hemoperitoneum. The typical pathophysiology of an obstructed appendiceal lumen resulting in appendiceal dilation and inflammation leading to suppuration and gangrene was absent. This unique presentation led to the initial diagnosis of appendiceal mass with appendicitis leading to an appendectomy. This was a near miss diagnosis had the abdominal cavity not been carefully explored after appendicectomy. We however recognize that a thorough abdominal cavity exploration should have been done before appendicectomy. Nevertheless, this would not have changed surgical management. Typically, imperforate hymen presents with primary amenorrhea and a cyclic pattern of lower abdominal/pelvic pain [7,8], with or without associated symptoms such as back pain (38%–40%), urine retention (37%–60%) [2,3], or constipation (27%). Blood accumulation in the vagina and uterus is evident when the distensible membrane bulges between the labia. However, blood flow dynamics for our case were different in that, retrograde blood flow through the right fallopian tube ended in the peritoneum. Consequently, no accumulation of blood to overly distend imperforate hymen (Fig. 1A). The inflamed appendicitis could have been caused by the irritation of blood in the peritoneum.

Summarized in Table 1 is the review of other case reports with both typical and atypical presentation of IH. Presentations include acute urinary retention, uterine infection and septic shock. Sixty percent of the cases presented with expanding abdominal mass and abdominal pain. In approximately 20%, the abdominal pain was cyclic, timing the menstruation period. Furthermore, a bluish bulging imperforate hymen, exaggerated on valsalva was a common finding on perineal examination. The vast majority of the clinical diagnosis was hematocolpometra. The amount of blood evacuated after hymenotomy ranged between 300–2500 mL, consistent with the amount of blood drained from our patient.

**Table 1:** Unusual and usual presentations of imperforate hymen.

Reference	Country	Age, y	Presentation	Abdominal exam	Vaginal Exam	Diagnosis	Surgical procedure	Volume of blood drained (mL)	Follow-up
[15]	USA	11	4-hour history of constant periumbilical pain that radiated to both flanks, associated with nausea, urinary frequency, and dysuria	Tenderness of the left lower quadrant and suprapubic region on palpation, with rebound and voluntary guarding	Imperforate hymen, which was vascular, bulging, bluish, and protruding from the introitus	Hematocolpometra	Hymenotomy	300	14-day follow-up visit, the patient reported that her symptoms had resolved
[16]	Germany	15	4-day history of increasing abdominal pain.	Nontender, no pulsatile midline mass extending from the pelvis to the umbilicus	Patient declined	Hematocolpometra	NR	NR	NR
[3]	UK	12	One-day history of acute urinary retention associated with suprapubic pain and dysuria	Soft with mild suprapubic and LIC tenderness	Nontender bluish-grey bulge posterior to the urethra	Hematocolpometra	Cruciate incision		Normal menses
[17]	Italy	13	Severe chronic pelvic pain which had started 1 year earlier	NR	NR, bulge not commented on	Pyocolpos	Incision, re-approximation of hymen edges with sutures	200 of pus	Regular menstrual cycles
[13]	Germany	14	Primary amenorrhea, expanding abdominal mass and abdominal pain	Nontender, soft, and homogenous mass, distorting her abdominal wall and expanding up to 5 cm over the umbilicus	Bulging forward	Hematocolpos	Oval shaped piece of hymen was excised, without suturing of the hymen remnant	2400	Recurrence of the hematocolpos, 2 months postop. A wider triangular tissue excision no recurrence after 12 months
[4]	India	14	Lower abdominal pain with abdominal distension	Large tender midline cystic mass noted extending from pelvis to epigastrium	Nontender bluish bulge	Hematocolpometra	Cruciate incision	2000	NR
[18]	Kenya	14	A week-long abdominal pains and tenesmus. No distension	Tender suprapubic mass corresponding to a uterus at 16 weeks	Bulging imperforate hymen, exaggerated on valsalva maneuvers. Rectal examination revealed an anterior mass	Hematocolpometra	X-shaped incision of the hymen. The edges of the hymen were everted and anchored by Vicryl 2/0 sutures	600	Doing well at one month
[7]	India	14	One-day history of colicky lower abdominal pain and acute retention of urine	Tender lower abdomen with a suprapubic mass corresponding to a uterus at 14 weeks	Bulging bluish color imperforate hymen, Exaggerated on valsalva maneuvers. Rectal examination revealed an anterior mass	Hematocolpometra	Cruciate incision. Mucosal margins everted and anchored by fine delayed absorbable suture (Vicryl 2/0)	800	Normal menses and patent outflow tract after one month
[7]	India	16	4-months history of colicky cyclical lower abdominal and mass	Tender, well defined, mobile, abdominal mass corresponds to 22 weeks gravid uterus with non-palpable lower border.	Bulged blush color Imperforated hymen	Hematocolpometra with hematosalpinx	X-shaped or cruciate incisions was made through the hymenal membrane at the 2-, 4-, 8-, and 10-o’clock. Margins of vaginal mucosa was approximated with fine delayed-absorbable suture		Normal menses and patent outflow tract after 1.5 months
[8]	Netherlands	16	One-year history of cyclical lower abdominal pain	Mobile, non-tender mass, arising from the pelvis to the umbilicus	Bluish bulging hymen	Hematocolpometra	Hymenectomy with a cruciate incision	500	Patient asymptomatic and started to have regular menstrual cycles, 2-month follow
[19]	Italy	3-day	Abdominal mass	Abdominal mass located in the mid region of the abdomen extending from the upper middle to the lowest region	Soft oval mass with an imperforate hymen	Hydrometrocolpos	Hymenectomy -incision of the hymenal membrane	100 milky fluids	Normal structure of the uterus
[20]	Turkey	8-month	Restlessness and intermittent fever of unknown etiology	Midline abdominal mass	Protruding imperforate hymen	Hydrometrocolpos	Simple cruciate incision was made over the hymen	500, cloudy, yellowish, non-bloody mucosal secretions	Gradual resolution of bilateral hydroureteronephrosis during 6-month follow-up
[21]	Australia	14	Severe left iliac fossa pain with her first episode of heavy bleeding per vagina	Marked lower abdominal tenderness on palpation, rebound tenderness and abdominal guarding	Partially perforated hymen with the rest of the hymen still intact.	Pyo-haemato-salpinges, hematometra, and hematocolpos	Cruciate incision I&D of right TOA	NR, purulent	6-weeks follow-up, good recovery with normal menses
[22]	USA	11	4-day history of lower abdominal and pelvic pain associated with palpable abdominal non-pulsatile mass that extended above the umbilicus	Palpable mass up to umbilicus	Bulging imperforate hymen		Vertical incision	2500	NR

***Abbreviations*:** NR: Not reported, I&D: Incision and drainage: TOA: Tubal ovarian abscess; USA; United States of America; UK; United Kingdom.

The diagnosis of IH can be made from a physical examination alone. Nevertheless, due to non-specific presentations, patients are usually investigated for other causes of abdomen pain, resulting to expensive laboratory testing or radiographic evaluations. Observing the hymen and viewing into the vaginal vault are best accomplished by applying gentle labial traction with the patient in the supine frog-leg or knee-chest position. In the typical presentation of IH with a bulging mass at the vaginal introitus, the speculum examination is not indicated. Failure to do a perineal examination such as in our case, leads to delayed or missed diagnosis. However, if patient or parents refuse genital exam evaluation, imaging studies can greatly help with diagnosis. Ultrasound scan will show an echogenic fluid accumulation in the vagina that can extend to uterus. In low-resources setting like ours, however, imaging modalities may not be available [9,10].

Although the presentation may be non-specific, once diagnosed, the treatment of imperforate hymen is either hymen sparing hymenotomy or non-sparing hymenectomy (excision of the hymenal tissue). The standard surgical procedure is hymenectomy using cruciate, T, plus, or X shaped (at 2-, 4-, 8-, and 10-o’clock positions) incisions and removal of excess hymenal tissue [11]. However, virginity sparing hymenotomy methods that involve application of a simple vertical incision without excising the hymen [2] or placement of a Foley catheter inserted in the vaginal introitus for 2 weeks [12] have also shown good outcome. One major advantage of a X shaped incision is the reduced risk of injury to the urethra but the disadvantage is the increased risk of refusion especially if suturing of the hymeneal edges is not performed, although the overall rate is generally low at 1% [13]. The procedure should be done with a urinary catheter in situ to prevent iatrogenic damage to the urethra. Application of uterine pressure to expel more blood is not advised as this practice is associated with retrograde flow of blood through the salpinx which could cause endometriosis and tubal adhesions. A laparoscopy may be needed for diagnosing intra-abdominal complications such as ruptured salpinx [14]. Finally, needle aspiration of hematocolpos is discouraged due to the risk of infection and pyocolpos formation.

Important and also actionable clinical implication of this case is improving accurate and timely diagnosis of imperforate hymen, particularly in those with atypical presentation. This calls for thorough genitourinary examination in girls of all ages from birth through the onset of menarche. Therefore, by performing examinations and by promptly diagnosing and treating imperforate hymen, we can prevent the incidence of obstructive symptoms and the associated complications due to delayed care.

In conclusion, this case suggests that imperforate hymen can mimic acute appendicitis. Gynecological history taking and careful external genital and introitus examination should be recommended in premenarchal girls presenting with abdominal pain. Imaging or laboratory studies are usually not indicated for a classical presentation of imperforate hymen. However, if a patient or parent refuses genital examination, imaging studies can greatly help with diagnosis.

## Funding

No funding for this study.

## Ethical approval

This article was approved by the ethics committee of the Eastern Regional Hospital, Koforidua, Ghana.

## Consent

Written informed consent was obtained from the patient’s parents as patient is minor, for publication of this case report and accompanying images. A copy of the written consent is available for review by the Editor-in-Chief of this journal on request.

## Author’s contribution

**Paddy Ssentongo:** Concept and design of study, data collection, data interpretation and analysis, drafting, revision, approval of final manuscript.

**Foster Amponsah:** Study design, performed surgery, data collection, revision, approval of final manuscript.

**Anna Ssentongo:** Data Collection, revision, approval of final manuscript.

**Temitope Arkorful:** Data collection, performed surgery, revision, approval of final manuscript.

**Seth Hansen-Garshong:** Data collection, performed surgery, revision, approval of final manuscript.

**Richard Ofosu-Akromah:** Data collection, performed surgery, revision, approval of final manuscript.

**John Oh:** Data interpretation, manuscript revision and final approval.

## Registration of research studies

Not applicable to our manuscript.

## Guarantor

Foster Amponsah-Manu.

## Provenance and peer review

Not commissioned, externally peer-reviewed.

## Declaration of Competing Interest

None declared. The authors have no financial, consultative, institutional, and other relationships that might lead to bias or conflict of interest.
